# Liver Transplantation for Quality as well as Quantity of Life

**DOI:** 10.5812/hepatmon.14890

**Published:** 2013-10-01

**Authors:** Kamran B Lankarani

**Affiliations:** 1 Health Policy Research Center, Shiraz University of Medical Sciences, Shiraz, IR Iran

**Keywords:** Liver Transplantation, Quality of Life, Patient Care

When we discuss various modalities of treatment, our aim is to measure the benefits. This benefit is usually defined as the years of saved life or increase the life expectancy in dreadful conditions. But life is not only the quantity. We know that the quality is even more important, but quantification of this outcome is not easy (1). There have been many proposed instruments for this aim. Now we know that as the quality of life is variously affected by the different diseases, we have to develop different quality measures for different diseases. This is especially more true for chronic diseases. Recipients of liver transplantation are at risk of special problems for instance rejection, long term expensive treatments, increased risk of cancer and opportunistic infections, increased rate of cardiovascular diseases and renal dysfunction. However without transplantation, they would have died within 12 months when their Model of end stage liver disease (MELD) score have raised to higher than 13. To monitor the quality of life after transplantation we have to consider all of these aspects (2).

Rule of culture, values, concerns and expectations from life in different cultures and even different stages of life should not be ignored when one consider the quality of life (3, 4). For instance, the quality of life for a Muslim may include her or his ability to go to mosque and pray which may not even discussed in other cultures. Sometimes expectations are not in line with medical advices and health life. Some people enjoy so much from smoking or other addiction that is considered a high quality of life in their opinion. However many aspects of quality of life is constant between peoples and cultures. Considering these backgrounds we should welcome the efforts of Tayebi and her colleagues in developing an instrument in measuring quality of life after liver transplantation in Iran (5). Their scientific research which is published in this issue of hepatitis monthly provided us with an instrument which could help clinicians as well as other health care providers for better care of these patients. They have noticed three areas with major effect on quality of life after liver transplantation: health satisfaction, concerns and complications. The content of their instrument was based on semi structured interview with real patients and literature review. The evaluated the face and content validity of their questionnaire through interview with liver transplant recipients and experts respectively and finally they evaluated construct as well as convergent validity and reliability of their instrument by interviewing 250 recipients. The final instrument after refinements had 39 items with Cronbach's alpha of 0.73 to 0.99 and correlation of 87 in the test-retest interval of two weeks for content, convergent and structure validity respectively. Despite these strengths several points about this report needs more considerations.

The indication of liver transplantation has important effect on quality of life after transplantation. Those suffering from chronic liver disease were usually in grave condition for months even years before transplantation with frequent hospitalization and low quality of life. Their improvement after transplantation is very promising and they usually enjoy a high quality of life specially in the first years after transplantation (6). Thereafter if long term complications are developed then the quality of life would change dramatically. There are very few diseases in which transplantation is a cure. For instance Wilson’s disease if without neurological complications, acute liver failure due to toxins and hereditary hypercholesterolemia are among these. But for most if the patients the liver transplantation is a modality of increasing life expectancy and quality with possibility of recurrence of disease even years after transplantation . Patients’ expectation should be built upon sound scientific basis. They should know that they have to use drugs even after transplantation and they should know about the recurrence and complications. Failure to disclose: these information may lead to low quality of life after transplantation because of unrealistic expectations.

Mental health of patients before and after liver transplantation also affects quality of life and should be considered in evaluation of post liver transplantation quality of life (7).

In my experience the patients’ physician relationship has a major effect on quality of life specially in chronic medical conditions .The health providers and in special the responsible physicians can help the patient to cope with the complications and can make their expectations more realistic even before transplantations. All of these have major effects on quality of life. This aspect is usually not well addressed in current instruments including the one proposed by Tayebi and her colleagues. We should say that this aspect is also very difficult to measure. One way might be to compare quality of life of patients between centers. Those centers with better quality of life may have reached this not only with higher experience in medical care and lower rates of medical complications but also with more professionalism and better psychological support of their patients.

Sexual life might not be as important for elderly but this affects the life of middle age and younger recipients deeply. This special issue needs to be well addressed (8). The costs of expensive treatments like transplantation and long term immunosuppressive treatment that these patients need also affect the recipients’ quality of life (9). This issue is not also considered in current models of measuring quality of life. Whether the donor was alive or cadaver and whether the recipient knows the identity of the donor may also change the quality of life (10). One threatening finding is that only 44% of liver recipients were employed. Liver transplantation should not lead to social isolation and early retirement. This should be a mode for leading to a productive life. This finding has high importance. It has shown recently that occupation and profession are of major concerns for the quality of life of patients in post transplantation period (8). The pre-operative consultations and preparation as well as post transplantation education and care should be adjusted to avoid unproductivity and unemployment.

Authors correctly pointed to the limitation of their study in having nonrandom samples. Extrapolation of their data to different patents from different backgrounds of diseases and cultures should be done with caution. We look forward for further prospective studies on this important issue in recipients of liver transplantation to have a better understanding of different dimensions of quality of life.

## References

[1] Higginson IJ, Carr AJ (2001). Using quality of life measures in the clinical setting.. BMJ..

[2] Chen PX, Yan LN, Wang WT (2012). Health-related quality of life of 256 recipients after liver transplantation.. World J Gastroentero..

[3] Tamburini MI, Higginson A, Carr P, Robinson (eds) . (2003). Quality of Life.. Ann Oncol..

[4] Werkgartner G, Wagner D, Manhal S, Fahrleitner-Pammer A, Mischinger H, Wagner M (2013). Long-term quality of life of liver transplant recipients beyond 60 years of age.. AGE..

[5] Tayebi Z, shahsavari H, Ebadi A, Parsa Z, Tayebi R, bolourchifard F (2013). Liver Transplant Recipients Quality of Life Instrument: Development and Psychometric Testing.. Hepat Mon..

[6] Perez-San-Gregorio MA, Martin-Rodriguez A, Dominguez-Cabello E, Fernandez-Jimenez E, Bernardos-Rodriguez A (2013). Quality of life and mental health comparisons among liver transplant recipients and cirrhotic patients with different self-perceptions of health.. J Clin Psychol Med S..

[7] Miller LR, Paulson D, Eshelman A, Bugenski M, Brown KA, Moonka D (2013). Mental health affects quality of life and recovery after liver transplantation.. Liver Transplant..

[8] Thiel C, Landgrebe K, Knubben E, Nadalin S, Ladurner R, Grasshoff C (2013). Contributors to individual quality of life after liver transplantation.. Eur J Clin Invest..

[9] Lankarani KB, Mahmoodi M, Gholami S, Mehravar S, Malekhosseini SA, Heydari ST (2012). Reducing Social Disparity in Liver Transplantation Utilization through Governmental Financial Support.. Hepat Mon..

[10] Togashi J, Sugawara Y, Akamatsu N, Tamura S, Yamashiki N, Kaneko J (2013). Quality of life after adult living donor liver transplantation: A longitudinal prospective follow‐up study.. Hepatol Res..

